# Case report: Early onset *de novo* FSGS in a child after kidney transplantation—a successful treatment

**DOI:** 10.3389/fped.2023.1280521

**Published:** 2023-09-26

**Authors:** Karla Carvajal Abreu, Sebastian Loos, Lutz Fischer, Lars Pape, Thorsten Wiech, Markus J. Kemper, Burkhard Tönshoff, Jun Oh, Raphael Schild

**Affiliations:** ^1^ Department of Pediatric Nephrology, Pediatric Hepatology and Pediatric Transplantation, University Medical Center Hamburg-Eppendorf, Hamburg, Germany; ^2^ Department of Hepatobiliary Surgery and Transplantation, University Medical Center Hamburg-Eppendorf, Hamburg, Germany; ^3^ Department of Pediatrics II, University Hospital of Essen, University of Essen-Duisburg, Essen, Germany; ^4^ Institute of Pathology, University Medical Center Hamburg-Eppendorf, Hamburg, Germany; ^5^ Department of Pediatrics, Asklepios Klinik Nord-Heidberg, Hamburg, Germany; ^6^ Department of Pediatrics I, University Children’s Hospital Heidelberg, Heidelberg, Germany

**Keywords:** *de novo* focal segmental glomerulosclerosis, kidney transplant, pediatric donor, proteinuria, plasmapharesis

## Abstract

**Background:**

Early onset *de novo* focal segmental glomerular sclerosis (FSGS) in the kidney allograft in patients without FSGS in the native kidney is a rare disorder in children. It usually occurs mostly beyond the first year after kidney transplantation and often leads to graft loss. Standardized treatment protocols have not yet been established.

**Case description:**

We describe a boy with early onset *de novo* FSGS in the transplanted kidney and non-selective glomerular proteinuria (maximum albumin-to-creatinine ratio of 3.8 g/g; normal range, ≤0.03 g/g creatinine). Manifestation occurred at 30 days posttransplant and was accompanied by a significant graft dysfunction (eGFR 61 ml/min per 1.73 m^2^). Treatment with 25 sessions of plasmapheresis over 14 weeks and three consecutive days of methylprednisolone pulse therapy (10 mg/kg per day) followed by oral prednisolone as rejection prophylaxis (3.73 mg/m^2^ per day) led to sustained remission of proteinuria (albumin-to-creatinine ratio of 0.028 g/g) and normalization of graft function (eGFR 92 ml/min per 1.73 m^2^) after 14 weeks. The follow-up period was 36 months.

**Conclusions:**

This case underlines the efficacy of immunosuppressive and antibody eliminating therapy in early onset *de novo* FSGS after kidney transplantation.

## Introduction

Focal segmental glomerular sclerosis (FSGS) in renal allografts can occur as recurrent disease or *de novo*, i.e., in patients without FSGS in the native kidney (1). In adults and children, the rate of recurrent FSGS is approximately 30% and is associated with a 50% risk of graft loss (2, 3). In contrast, *de novo* FSGS after kidney transplantation is a rare condition occurring in 0.6% to 1.6% of adult transplant recipients (4, 5). Research on children with *de novo* FSGS after kidney transplantation is limited.

Recurrent FSGS is thought to be mediated by soluble permeability factors that permeabilize the filtering glomerular unit leading to glomerular injury (6, 7). While treatment is based on enhanced immunosuppressive therapy and removal of these putative permeability factors by plasmapheresis, the pathogenesis of *de novo* FSGS is unknown (7–9). Secondary causes are thought to be predominant in *de novo* FSGS (5). These include hyperfiltration injury due to low nephron number (e.g., due to donor-recipient size mismatch), calcineurin inhibitor (CNI) toxicity, sirolimus treatment, and specific viral infections such as SARS-COV-2, HIV, and parvovirus (5, 6, 10, 11). Due to the suspected secondary nature of the condition, there is no established evidence-based treatment protocol. Treatment is highly dependent on the cause of *de novo* FSGS posttransplant and consists of renin-angiotensin-aldosterone system (RAAS) inhibition, reduction of CNI exposure, or treatment of the underlying viral infection. However, in a subset of *de novo* FSGS cases, the pathogenesis is thought to be similar to primary idiopathic FSGS in the native kidney, and the condition is often referred to as primary idiopathic *de novo* FSGS (12, 13). In these cases, treatment strategies are like those for primary or recurrent FSGS and consist of intensified immunosuppressive and antibody depleting therapy, including cyclophosphamide, rituximab, and plasmapheresis (14).

We present a young male who developed *de novo* FSGS after kidney transplantation, which was successfully treated with plasmapheresis for three months in combination with methylprednisolone pulse therapy.

## Case report

The patient is a 10-year-old male of German-Egyptian origin, whose primary kidney disease was posterior urethral valves. Bilateral hydronephrosis and oligohydramnios were noted during pregnancy. He was born by emergency cesarean section at a gestational age of 37 weeks. Signs of chronic kidney disease were present at birth. At 4 years of age, a nephroureterectomy of the non-functioning left kidney was performed due to recurrent urinary tract infections. Kidney function deteriorated during early childhood and hemodialysis was started at the age of 9 years.

After two years of hemodialysis, the patient received his first ABO compatible kidney transplant from a deceased pediatric donor aged 2 years (12 kg body weight, drowning victim). While in the intensive care unit and prior to donation, the donor had developed mild proteinuria and glucosuria due to moderate hyperglycemia (364 mg/dl). There were 4 HLA mismatches (1 HLA-A, 2 HLA-B, and 1 HLA-DR mismatch). Posttransplant the patient developed a mildly delayed graft function requiring a single hemodialysis session due to hyperphosphatemia and hyperkalemia. Kidney function parameters normalized to a serum creatinine of 0.9 mg/dl after 14 days. Since postoperative day 2, the patient developed persistent arterial hypertension, which was treated with metoprolol succinate and amlodipine. The immunosuppressive regimen consisted of prednisolone, mycophenolate mofetil (1,200 mg/m^2^ per day) and tacrolimus (initial target trough levels: between 8 and 10 µg/L) according to our local standard. No induction therapy was used according to local standards. On the day of discharge (day 20 posttransplant), the serum creatinine level was 1.10 mg/dl, corresponding to an estimated glomerular filtration rate (eGFR) of 52 ml/min per 1.73 m^2^.

Since day 28 posttransplant, the patient developed progressive non-selective nephrotic-range glomerular proteinuria [maximum albumin-to-creatinine ratio (ACR) of 3.8 g/g] with mild hypoalbuminemia (32 mg/L) and microscopic hematuria with initially stable graft function. On day 38 posttransplant, he was admitted to the hospital for a kidney transplant biopsy. His blood pressure was markedly elevated. There was no evidence of peripheral edema or fever. The patient complained of right upper and lower quadrant tenderness on the same side as the graft. Laboratory tests showed no evidence of infection.

**Figure 1:**
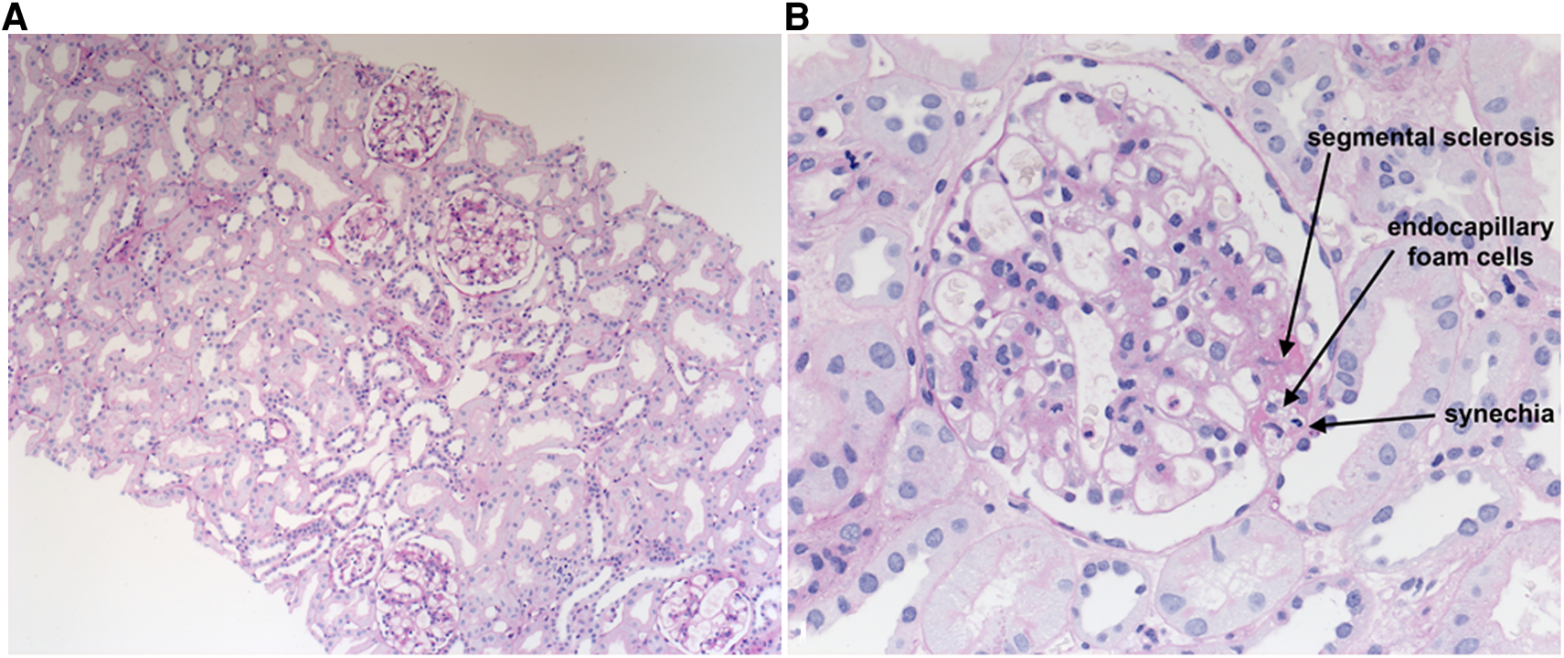
Histological lesions in kidney allograft with *de novo* FSGS. Description: Several enlarged glomeruli demonstrate FSGS lesions that were predominantly tip lesions with endocapillary foam cells on PAS staining (**A,B**). Prof. Dr. med. Wiech, Institute of Pathology, University Medical Center Hamburg-Eppendorf, 2017.

Histology of the kidney transplant showed FSGS in 9 of 33 glomeruli characteristic of primary FSGS. Two of the 9 glomeruli were completely sclerosed and 7 of the 9 glomeruli showed segmental sclerosis mostly involving the glomerular tip (“tip lesions”) with endocapillary foam cells and small capsular synechiae (Figure 1). No collapsing or perihilar lesions were observed. Immunofluorescence examination showed no deposits of IgA, IgG, C1q, C3, IgM, or fibrinogen/fibrin. Peritubular capillaries were C4d negative. Electron microscopy showed glomerular capillaries with partial loss of podocyte foot processes with focally loosened inner basement membrane layers without splintering or rupture. No electron dense deposits were observed.

Serologic testing and blood PCR analysis revealed no evidence of ongoing infection with herpes simplex virus, Epstein-Barr virus, cytomegalovirus, BK polyomavirus, or hepatitis B or C, or parvovirus B19. Due to a decline in kidney transplant function over the following 10 days (eGFR 82 to 61 ml/min per 1.73 m^2^), we administered methylprednisolone pulse therapy (10 mg/kg per day for three consecutive days) followed by oral prednisolone (Graphic 1). Simultaneously plasmapheresis (2,500 ml exchange volume, 1.5 plasma volume, albumin as replacement fluid) was administered on day 67 posttransplant. Initially, plasmapheresis was administered daily for one week and gradually tapered to twice weekly after 3 weeks and once weekly after 7 weeks; treatment was stopped after 14 weeks and a total of 25 sessions.

**GRAPHIC 1:**
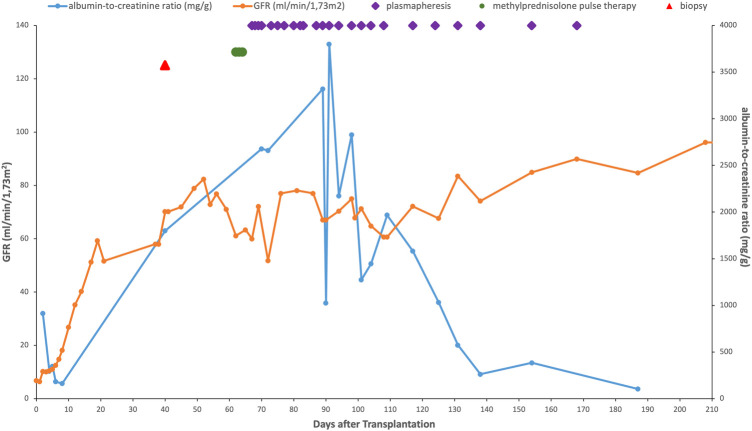
Evolution of the GFR, albumin-to-creatinine ratio and successive treatments of *de novo* early-onset posttransplant FSGS in the patient after transplantation. Reference ranges: eGFR ≥90 ml/min per 1.73 m^2^, albumin-to-creatinine ratio (ACR) ≤ 30 mg/g creatinine

Partial remission of proteinuria (ACR 0.57 g/g) was observed after 7 weeks of plasmapheresis and complete remission (ACR 0.12–0.34 g/g) after 14 weeks. At the end of treatment, eGFR improved substantially during plasmapheresis from 61 ml/min per 1.73 m^2^ to 93 ml/min per 1.73 m^2^. During the 36-month follow-up period, sustained remission of proteinuria and further improvement in graft function were observed (ACR at 36 months posttransplant, 28 mg/g; eGFR, 92 ml/min per 1.73 m^2^), while hypertension remained in the 80th percentile.

## Discussion

We present a case of very early onset *de novo* FSGS after kidney transplantation in a young male who was successfully treated with plasmapheresis in combination with methylprednisolone pulse therapy. In contrast to recurrent FSGS, the development of *de novo* FSGS is rare (5). The time of onset is often more than 12 months after transplantation and a progressive deterioration of graft function is common (5, 9). Upon initiation of therapy, our patient presented with worsening kidney graft function, increasing nephrotic-range proteinuria, and mild hypalbuminemia without peripheral edema.

While the etiology of *de novo* FSGS remains unclear, there are predisposing factors that may be associated with an increased risk of developing *de novo* FSGS: CNI toxicity, mTOR inhibitor therapy (e.g., sirolimus), rejection, and conditions that lead to glomerular hyperfiltration by reducing the number of nephrons (15, 16). These predisposing factors include diabetes mellitus, arterial hypertension, and infections such as parvovirus B19 or BK polyomavirus (5). Since 2019, COVID-19 has emerged as a new cause of *de novo* glomerulopathies, causing mainly collapsing but also non-collapsing FSGS in native or transplanted kidneys (17). Furthermore, a size mismatch between the kidney allograft and the recipient's body mass can lead to glomerular hyperfiltration due to a relatively low number of nephrons (nephron underdosing) (9). Of these risk factors, treatment with a CNI, arterial hypertension, and a relative size mismatch were present in our patient. However, there was no evidence of typical histologic features of CNI nephrotoxicity such as arteriolar hyalinosis or striped interstitial fibrosis/tubular atrophy (5). Therefore, CNI toxicity most likely did not contribute to the development of FSGS in this patient. There was no evidence of graft rejection or any other form of glomerulonephritis or polyomavirus nephropathy.

The young age of the kidney donor may have put our patient at risk for hyperfiltration injury. However, size mismatch is typically more pronounced in adult recipients of small pediatric organs. In the pediatric recipient, the size mismatch was observed to be moderate. In addition, secondary FSGS due to arterial hypertension/hyperfiltration injury is typically characterized by perihilar localization of FSGS lesions (18). However, the “tip lesions” seen in our patient are characteristic of primary FSGS (19). Hyperfiltration and hypertension may have contributed to the glomerular lesions, but we consider it unlikely that our patient developed *de novo* FSGS solely due to these factors alone.

An unrecognized primary genetic form of FSGS in the donor may have contributed to our patient's condition. The second kidney from the same donor was transplanted to a young male in Germany who also developed mild proteinuria (max ACR 0.46 g/g) and graft dysfunction. On day 38 posttransplant, a kidney transplant biopsy revealed severe acute antibody-mediated rejection (ABMR). 1 of 30 glomeruli showed evidence of segmental sclerosis. Proteinuria resolved after rejection treatment. These findings in the second recipient, together with the response to plasmapheresis in our recipient, do not support a diagnosis of genetic FSGS in the donor. While it is possible that other donor factors, such as the young donor age, may have played a role, we consider it is likely that ABMR was the primary cause of proteinuria in the second recipient.

Based on the histology, the early onset, and the lack of convincing evidence for secondary forms, we suspected primary idiopathic *de novo* FSGS in our patient. Therefore, we started a more intensive immunosuppressive therapy with methylprednisolone pulses in combination with plasmapheresis. This therapy has been proposed as initial therapy for recurrent FSGS in the transplanted kidney and is widely used for severe forms of primary FSGS in the native kidney (8, 9). As an anti-CD20 antibody, rituximab has been shown to induce partial or complete remission in a fraction of patients with primary FSGS in the native kidney and its recurrence after kidney transplantation (11, 20–22). Therefore, we proposed a course of rituximab, but the parents did not give consent because of concerns about the risk of infection.

Our case report suggests that plasmapheresis in combination with methylprednisolone is an effective treatment for *de novo* FSGS in the transplanted kidney, even without rituximab therapy. However, there is evidence that the outcome of the tip variant is superior to the other histologic variants in the native kidney (23). Spontaneous improvement may have played a role in the patient's remission, but a complete spontaneous remission of *de novo* FSGS is extremely rare (24). Therefore, due to the severity of the disease and the overall high risk of graft loss, we strongly advocate aggressive treatment of this disease at least in the presence of graft dysfunction.

This report is limited by the lack of an initial intraoperative allograft biopsy and the inherent uncertainties regarding the relationship between histopathologic classification and disease etiology. Further studies investigating the outcome and management of *de novo* FSGS after kidney transplantation in pediatric recipients are urgently needed to further guide the therapeutic management of these patients.

## Data Availability

The raw data supporting the conclusions of this article will be made available by the authors, without undue reservation.

## References

[1] Abbas F El Kossi M Jin JK Sharma A Halawa A. De novo glomerular diseases after renal transplantation: how is it different from recurrent glomerular diseases? World J Transplant. (2017) 7(6):285–300. 10.5500/wjt.v7.i6.285 29312858 PMC5743866

[2] Chadban S. Glomerulonephritis recurrence in the renal graft. J Am Soc Nephrol. (2001) 12(2):394–402. 10.1681/ASN.V122394 11158232

[3] Artero M Biava C Amend W Tomlanovich S Vincenti F. Recurrent focal glomerulosclerosis: natural history and response to therapy. Am J Med. (1992) 92(4):375–83. 10.1016/0002-9343(92)90267-F 1558084

[4] Hariharan S Adams MB Brennan DC Davis CL First MR Johnson CP Recurrent and de novo glomerular disease after renal transplantation: a report from renal allograft disease registry (RADR). Transplantation. (1999) 68(5):635–41. 10.1097/00007890-199909150-00007 10507481

[5] Patel RD Vanikar AV Nigam LA Kanodia KV Suthar KS Patel HV. De novo focal segmental glomerulosclerosis in renal allograft-histological presentation and clinical correlation: single centre experience. J Clin Diagn Res. (2017) 11(4):Ec39–ec42. 10.7860/JCDR/2017/25502.9728 28571148 PMC5449794

[6] Vincenti F Angeletti A Ghiggeri GM. State of the art in childhood nephrotic syndrome: concrete discoveries and unmet needs. Front Immunol. (2023) 14:1167741. 10.3389/fimmu.2023.1167741 37503337 PMC10368981

[7] Kang HG Ha IS Cheong HI. Recurrence and treatment after renal transplantation in children with FSGS. BioMed Res Int. (2016) 2016:6832971. 10.1155/2016/6832971 27213154 PMC4860214

[8] Shishido S Satou H Muramatsu M Hamasaki Y Ishikura K Hataya H Combination of pulse methylprednisolone infusions with cyclosporine-based immunosuppression is safe and effective to treat recurrent focal segmental glomerulosclerosis after pediatric kidney transplantation. Clin Transplant. (2013) 27(2):E143–50. 10.1111/ctr.12079 23383697

[9] Ponticelli C Moroni G Glassock RJ. De novo glomerular diseases after renal transplantation. Clin J Am Soc Nephrol. (2014) 9(8):1479–87. 10.2215/CJN.12571213 24700797 PMC4123406

[10] Levenson E Shepherd TN Aviles D Craver R Ehlayel A Love GL De novo collapsing glomerulopathy in a pediatric kidney transplant recipient with COVID-19 infection. Pediatr Transplant. (2021) 25(4):e14013. 10.1111/petr.14013 33773007

[11] Lim WH Shingde M Wong G. Recurrent and de novo glomerulonephritis after kidney transplantation. Front Immunol. (2019) 10:1944. 10.3389/fimmu.2019.01944 31475005 PMC6702954

[12] Stokes MB Davis CL Alpers CE. Collapsing glomerulopathy in renal allografts: a morphological pattern with diverse clinicopathologic associations. Am J Kidney Dis. (1999) 33(4):658–66. 10.1016/S0272-6386(99)70216-7 10196006

[13] Meehan SM Pascual M Williams WW Tolkoff-Rubin N Delmonico FL Cosimi AB De novo collapsing glomerulopathy in renal allografts. Transplantation. (1998) 65(9):1192–7. 10.1097/00007890-199805150-00009 9603167

[14] Han KH Kim SH. Recent advances in treatments of primary focal segmental glomerulosclerosis in children. BioMed Res Int. (2016) 2016:3053706. 10.1155/2016/3053706 27195285 PMC4852325

[15] Letavernier E Bruneval P Mandet C, Duong Van Huyen JP Peraldi MN Helal I High sirolimus levels may induce focal segmental glomerulosclerosis de novo. Clin J Am Soc Nephrol. (2007) 2(2):326–33. 10.2215/CJN.03751106 17699432

[16] Ivanyi B. A primer on recurrent and de novo glomerulonephritis in renal allografts. Nat Clin Pract Nephrol. (2008) 4(8):446–57. 10.1038/ncpneph0854 18560395

[17] Klomjit N Zand L Cornell LD Alexander MP. COVID-19 and glomerular diseases. Kidney Int Rep. (2023) 8(6):1137–50. 10.1016/j.ekir.2023.03.016 37274308 PMC10041821

[18] Harvey JM Howie AJ Lee SJ Newbold KM Adu D Michael J Renal biopsy findings in hypertensive patients with proteinuria. Lancet. (1992) 340(8833):1435–6. 10.1016/0140-6736(92)92624-O 1360561

[19] Howie AJ Brewer DB. The glomerular tip lesion: a previously undescribed type of segmental glomerular abnormality. J Pathol. (1984) 142(3):205–20. 10.1002/path.1711420308 6707787

[20] Garrouste C Canaud G Büchler M Rivalan J Colosio C Martinez F Rituximab for recurrence of primary focal segmental glomerulosclerosis after kidney transplantation: clinical outcomes. Transplantation. (2017) 101(3):649–56. 10.1097/TP.0000000000001160 27043407

[21] Kronbichler A Kerschbaum J Fernandez-Fresnedo G Hoxha E Kurschat CE Busch M Rituximab treatment for relapsing minimal change disease and focal segmental glomerulosclerosis: a systematic review. Am J Nephrol. (2014) 39(4):322–30. 10.1159/000360908 24751753

[22] Trautmann A Vivarelli M Samuel S Gipson D Sinha A Schaefer F IPNA Clinical practice recommendations for the diagnosis and management of children with steroid-resistant nephrotic syndrome. Pediatr Nephrol. (2020) 35(8):1529–61. 10.1007/s00467-020-04519-1 32382828 PMC7316686

[23] D'Agati VD Kaskel FJ Falk RJ. Focal segmental glomerulosclerosis. N Engl J Med. (2011) 365(25):2398–411. 10.1056/NEJMra1106556 22187987

[24] Deegens JK Assmann KJ Steenbergen EJ Hilbrands LB Gerlag PG Jansen JL Idiopathic focal segmental glomerulosclerosis: a favourable prognosis in untreated patients? Neth J Med. (2005) 63(10):393–8. 16301760

